# Sleep Phases in Crayfish: Relationship Between Brain Electrical Activity and Autonomic Variables

**DOI:** 10.3389/fnins.2021.694924

**Published:** 2021-10-14

**Authors:** Mireya Osorio-Palacios, Laura Montiel-Trejo, Iván Oliver-Domínguez, Jesús Hernández-Falcón, Karina Mendoza-Ángeles

**Affiliations:** Laboratorio de Redes Neuronales, Departamento de Fisiología, Facultad de Medicina, Universidad Nacional Autónoma de México (UNAM), México City, Mexico

**Keywords:** invertebrate, behavior, electrophysiology, wavelet transform, unsupervised learning techniques, Pearson correlation matrix

## Abstract

In vertebrates like mammals and birds, two types of sleep have been identified: rapid eye movement and non-rapid eye movement sleep. Each one is associated with specific electroencephalogram patterns and is accompanied by variations in cardiac and respiratory frequencies. Sleep has been demonstrated only in a handful of invertebrates, and evidence for different sleep stages remains elusive. Previous results show that crayfish sleeps while lying on one side on the surface of the water, but it is not known if this animal has sleep phases. Heart rate and respiratory frequency are modified by diverse changes in the crayfish environment during wakefulness, and previously, we showed that variations in these variables are present during sleep despite that there are no autonomic anatomical structures described in this animal. Here, we conducted experiments to search for sleep phases in crayfish and the relationships between sleep and cardiorespiratory activity. We used the wavelet transform, grouping analysis with *k*-means clustering, and principal component analysis, to analyze brain and cardiorespiratory electrical activity. Our results show that (a) crayfish can sleep lying on one side or when it is motionless and (b) the depth of sleep (measured as the power of electroencephalographic activity) changes over time and is accompanied by oscillations in cardiorespiratory signal amplitude and power. Finally, we propose that in crayfish there are at least three phases of sleep.

## Introduction

Sleep is generally defined as a rapidly reversible state of immobility and greatly reduced sensory responsiveness, characterized by a species-specific posture (Flanigan et al., 1974). A further criterion is that sleep is homeostatically regulated; the loss of sleep is followed by an increased need for sleep and a consequent “sleep rebound” (Tobler, 2005). In vertebrates like mammals and birds, distinctive electrophysiological patterns accompany behavioral sleep, which may be divided into rapid eye movement (REM) and non-rapid eye movement (NREM) sleep. REM sleep is associated with low-voltage and high-frequency electroencephalographic (EEG) activity, nearly indistinguishable from the activity observed during wakefulness. Non-rapid eye movement sleep is divided into light sleep (slowing of the EEG waves), and deep slow-wave sleep (SWS) (characterized by high-amplitude slow waves). Non-rapid eye movement EEG activity varies depending on sleep depth, but it is generally slower and of higher amplitude than the EEG activity observed during either wake or REM sleep (Rechtschaffen and Kales, 1968). Studies have demonstrated that a variety of physiological changes take place during the different stages of sleep. NREM sleep is characterized by decreases in muscle tone, body movements, heart rate (HR), respiratory frequency (RF), blood pressure (BP), metabolic rate, and temperature. These parameters reach their lowest values during SWS. By contrast, REM sleep is accompanied by increases in blood pressure, HR, RF, and metabolism to levels almost as high as those found in the awake state (Foulkes and Schmidt, 1983; Trinder et al., 2001). Although most evidence suggests that sleep is related to many diverse processes such as memory consolidation, emotional stability, and maintenance of brain homeostasis, the mechanisms by which sleep fulfills these functions are unclear (Rechtschaffen, 1998; Maquet, 2001; Siegel, 2003, 2005; Frank, 2006; Cirelli and Tononi, 2008; Walker, 2009, 2010; Diekelmann and Born, 2010; Schmidt, 2014; Yamazaki et al., 2020). Most sleep researchers accept the idea that the function of NREM sleep is, at least in part, restorative and SWS is specifically involved in maintaining synaptic homeostasis in mammals (Tononi and Cirelli, 2003, 2006). Nevertheless, no definitive answer has yet been found to the main question: what is the function of sleep?

A different approach to discovering the origins and functions of sleep would be through the study of non-mammalian organisms that have remained relatively unchanged from their ancient fossil ancestors and which may provide clues about sleep. There is general agreement that most non-mammalian organisms exhibit behavioral sleep. However, the electrophysiological signs of sleep in these organisms may be very different from that of mammals (Hartse, 2011).

**FIGURE 1:**
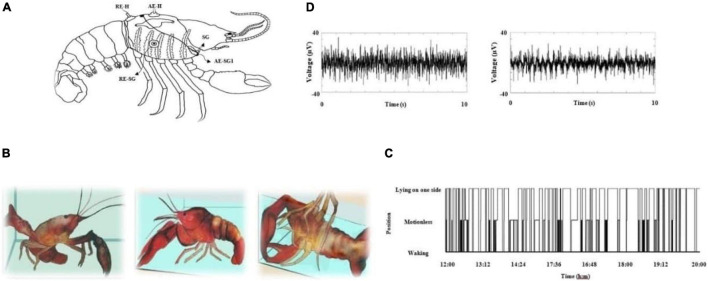
**(A)** Schematic representation of electrode placement to obtain electrophysiological recordings. On the dorsal carapace, two electrodes span the dorsal–caudal axis of the heart and monitor cardiac electrical activity. Two hook electrodes (one on each gill chamber) monitor the electrical activity generated by the scaphognathites (SG); a third electrode (RE-SG) was the reference electrode for these structures. RE-H (reference electrode-heart), AE-H (active electrode-heart), AE-SG1 (active electrode-scaphognathites side1). For the brain surface, we implanted with Teflon-coated Pt-Ir electrodes (75 μm diameter) on deutocerebrum (see Hernández et al., 1996). **(B)** Crayfish in several positions: alert (left panel), motionless (middle panel) and lying on one side (right panel) crayfish. **(C)** Crayfish position during 8 continuous hours and **(D)** brain electrical activity in a behaving freely crayfish; alert (left panel) and lying on one side (right panel).

Crayfish is an animal with a relatively small number of neurons in its brain, and this is the only invertebrate, in which sleep has been described based on the same kind of behavioral and electrophysiological criteria defined for vertebrates and, in an unrestrained and complete animal (Ramón et al., 2004). Sleep in crayfish is characterized by a stereotypical position (lying on one side against the surface of water, Figure 1B), increase in sensory threshold, absence of electrical signs of cognitive processes, a brain electrical activity unique for this state, and a strong decrease in power at frequencies about 30–45 Hz. During wakefulness, crayfish walks around the aquarium and recordings from the brain surface show numerous spikes of various sizes superimposed on an almost flat baseline. In the sleeping animal, the spikes are substituted by slow waves with frequencies ranging from 10 to 20 Hz (Ramón et al., 2004; Mendoza-Ángeles et al., 2010). No sleep phases have been described for this or any other invertebrate. However, it is postulated that in *Drosophila* there are sleep stages (van Alphen et al., 2013).

In some crustaceans, as in crayfish, it is known that aggressive and submissive social interactions, copulation, and perturbations in the environment induce changes in heartbeat and respiration (Schapker et al., 2002; Shuranova et al., 2006; Cooper et al., 2011; Canero and Hermitte, 2014). Previously, we showed for the first time that changes in these variables occur during sleep, too (Osorio-Palacios et al., 2021). However, there are no descriptions of an autonomic nervous system; we ignore the mechanisms and pathways mediating this regulation, but it is quite conspicuous.

The main purpose of this work was to analyze physiological time series from crayfish’s brain and cardiorespiratory electrical activity by wavelet transform, Pearson correlation matrix and unsupervised learning techniques, in order to search for sleep phases and determine the relationship between these activities during sleep.

## Materials and Methods

### Animals

We used adult male crayfish *Procambarus clarkii* (7–10 cm rostrum to tail), 20–30 g dry weight, obtained from a local provider. Since their arrival to the laboratory, they were maintained in individual aquaria (30 × 15 × 10 cm with 5 cm of well-oxygenated tap water) at ambient temperature (20°C) under 12-h light–dark cycles (lights on at 07:00 and lights off at 19:00) and fed twice a week with cat chow. We used unrestrained animals in intermolt (*n* = 3).

### Behavioral Recordings

To determine crayfish positions, we videotaped individual animals continuously during periods of 8 h with a video camera (Sony, CX405, Mexico), and simultaneously recorded the electrophysiological activity. Experiments were conducted between 12:00 and 20:00 h.

### Electrophysiological Recordings

To determine the relationships between crayfish’s brain states and the cardiorespiratory activity, we implanted electrodes in the following structures: brain, pericardial sinus, and both-gill chambers.

#### Brain Electrical Activity

To record the brain electrical activity, we followed the procedure described elsewhere (Ramón et al., 2004). Briefly, we drilled a hole on the dorsal carapace of cold-anesthetized crayfish to introduce a stainless-steel cannula (Lanceta HG, Mexico) of 1-mm external diameter, carrying two platinum–iridium (Pt–Ir) Teflon-coated wires (A-M Systems, 787000, Sequim, WA, United States) of 75-μm diameter. Under stereoscopic microscopic control (Zeiss, OPMI-MD, Jena, Germany), we descended the cannula using a micromanipulator (Sutter Instrument, MM-33/R, Novato, CA, United States) and placed it on the deutocerebrum (Mendoza-Ángeles et al., 2010). Then, we fixed the cannula to the cephalothorax with dental cement (Acrimin Autocurable, Mexico); the cannula was used as a reference electrode.

#### Cardiac Electrical Activity

To obtain cardiac electrical activity, we made a hole at the level of the pericardial sinus of cold-anesthetized crayfish. Through this hole, we introduced a PVC tube (Intramedic, 7426, Sollentuna, Sweden) carrying a Pt–Ir Teflon-coated wire (A-M Systems, 787000, United States), 75 μm in diameter and 2 mm in length, and fixed the ensemble to the exoskeleton with dental cement (Acrimin Autocurable, Mexico). This electrode was used as an active one. Another electrode was introduced 5 mm away from the active one (rostrum to tail) and was used as a reference electrode. The disposition of electrodes is illustrated in Figure 1A.

#### Respiratory Electrical Activity

Gills are common breathing structures among aquatic invertebrates that allow them to exchange oxygen and CO_2_ with surrounding water (Bush et al., 1987). In crayfish, the gills are located inside the branchial chambers (Díaz and Rodriguez, 1977; Barra et al., 1983; Swain et al., 1988) beneath the exoskeleton and are attached to the bases of both the walking and claw-bearing legs. Each gill chamber is ventilated by a paired respiratory pump in the form of beating scaphognathites (Zoond and Charles, 1931; Hughes et al., 1969; Dyer and Uglow, 1978; Decelle et al., 2010).

To record the electrical activity generated by the muscles controlling the scaphognathites, we placed a hook electrode into each of the gill chambers of cold-anesthetized crayfish (active electrodes) and introduced a third electrode through the lateral region of the exoskeleton (reference electrode). We fixed the electrode wires to the carapace with dental cement (Acrimin Autocurable, Mexico); see Figure 1A. All electrodes were Pt–Ir Teflon-coated wires (A-M Systems, 787000, WA, United States) 75 μm in diameter.

### Signal Acquisition

Once crayfish were implanted, they were returned to their individual aquaria and left undisturbed for at least 24 h. Then, we videotaped and recorded the brain and cardiorespiratory electrical activities from isolated crayfish during 8 continuous hours (start at 12:00 h, end at 20:00 h).

The brain electrical activity was bandpass filtered between 3 Hz and 3 kHz; a 60-Hz notch filter was sometimes used. Electrical signals were preamplified with AC amplifiers (CWE, BM400, United States) and in parallel sampled at 2 kHz by an A/D converter (National Instruments, NI-USB-6211, Austin, TX, United States).

The cardiorespiratory electrical activity was band-pass filtered between 1 Hz and 1 kHz and sampled at 100 Hz. We acquired all data using a MATLAB software (MathWorks)-based algorithm developed in our laboratory and stored on a personal computer for off-line analysis. The experiments were approved by the Ethical Committee of the Faculty of Medicine at UNAM 023/2018.

### Data Analysis

#### Behavioral Analysis

To analyze crayfish behavior recordings, we defined three conditions of the animal: walking, lying on one side, and motionless. We associated each behavioral condition with the recording time. In the first condition, the animal walks around the aquarium, touches the walls with their long antennae, and eventually stays motionless or lying on one side. This last position has been described thoroughly in previous papers (Ramón et al., 2004; Mendoza-Ángeles et al., 2007, 2010). It consists in an animal resting with the legs of one side supporting the weight of the crayfish at the bottom of the aquarium while the contralateral legs are placed on the wall of the aquarium. In this way, both chelae are suspended and hang to the bottom of the aquarium. Finally, the last position consists simply in a motionless crayfish with both chelae resting on the bottom; see these different positions in Figure 1B.

We elaborated a position versus time graph from these data for the whole period of 8 continuous hours of monitoring, marking the duration of each condition and the instants of time where a transition from one condition to another occurred. Then, we associated each position identified with the electrophysiological recordings.

#### Electrophysiological Analyses

Once having identified the crayfish’s different positions adopted along the behavioral recording, we only selected those segments in which it was at least 5 continuous minutes lying on one side (we considered each one of these as a complete sleeping episode) or motionless.

We analyzed at least 25 examples of complete sleeping episodes corresponding to the three crayfish.

The duration of the analyzed sleeping episodes varies between 5 and 20 min. For each one, brain and cardiorespiratory electrical activities were partitioned in segments of 30 s and analyzed by wavelet transform (WT). We determined the temporal patterns of sleep over time by Pearson correlation matrix.

Finally, *k*-means clustering and principal component analysis (PCA) were used to determine the relationship between all variables.

From the complete recording shown in Figure 1C, a total of 16 complete sleeping episodes were selected. Figure 5 shows one of these episodes, which comprises 37 segments, each lasting 30 s, and are consecutive during the complete sleeping episode. We also include 1 min before and one after each sleeping episode.

**FIGURE 2:**
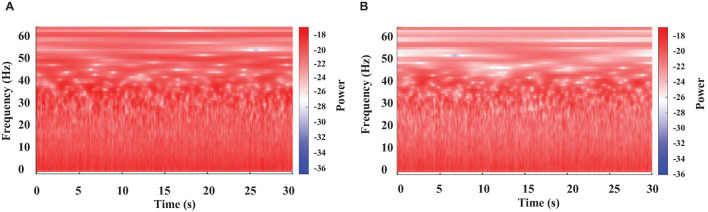
Color-coded representation of the WT results obtained from brain electrical activity in a crayfish alert **(A)** or lying on one side **(B).**

**FIGURE 3:**
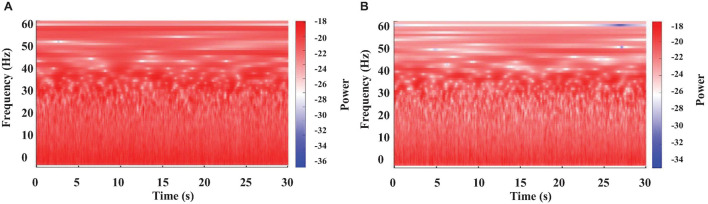
Color-coded representation of the WT obtained from a crayfish brain electrical activity at two different moments of a long motionless period. One minute after starting to be motionless **(A)**, and 2 min later **(B)**.

**FIGURE 4:**
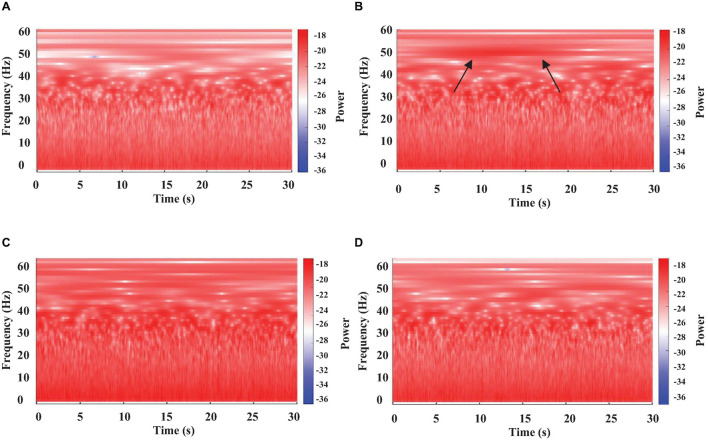
Color-coded representation of the WT of brain electrical activity from four different segments (**A–D** panels) of a single long-lasting episode of sleep in a crayfish lying on one side. Arrows in **(B)** panel indicate a micro-state with high power.

**FIGURE 5:**
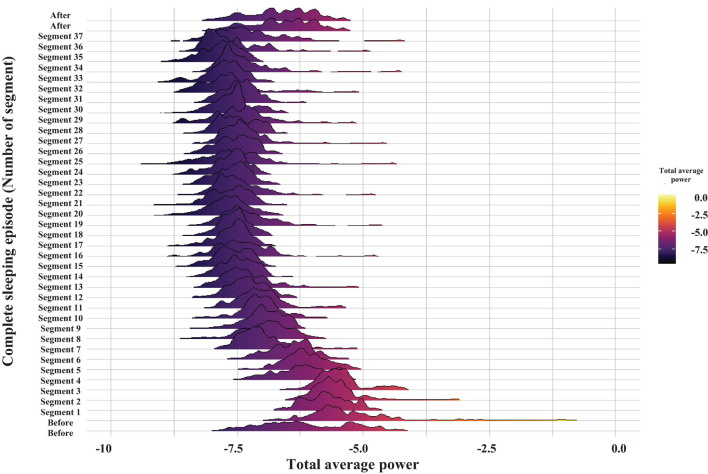
Probability distribution of the total average power of brain electrical activity for the frequency band of 30–60 Hz. The graph displays the time evolution of the probability distribution over a complete sleeping episode of about 20 min from bottom to top. Each segment shows the probability distribution of total average power during the 30 s windows. The beginning of the sleeping episode corresponds to segment number 1, and it finishes at segment number 37. The figure also includes a minute before and one after the sleeping episode. The power values were obtained by the WT analysis and are coded in color (high powers—light tones and low powers—dark tones).

In the case of a motionless crayfish, only brain electrical activity was analyzed by WT. The corresponding results are represented in Figure 3.

##### Wavelet Transform

Wavelet transform (WT) is a linear time–frequency representation that preserves time shifts and time scaling, which is appropriate for detection of transients in non-stationary signals. To perform a WT, we use a mother wavelet which, as the name suggests, is a localized waveform. Mathematically, the WT may be interpreted as a convolution of a signal with the mother wavelet, which is automatically adjusted to different time scales, that is, getting longer for slow and smaller for large frequency components (Addison, 2005).

We used the WT to analyze brain and cardiorespiratory activities during all complete sleeping episodes. First, we divided each sleeping episode in 30 s segments. For brain electrical activity, we used a software developed in our laboratory using MATLAB. For cardiorespiratory activities, we used the wavelet toolbox. In both cases, cardiac and respiratory activities, we used the Morlet wavelet.

WT analysis allowed us to obtain main frequencies and power for different frequency bands in brain, cardiac, and respiratory activities.

For brain electrical activity, only the 0-60 Hz band of interest is considered, as previously described (Ramón et al., 2004; Mendoza-Ángeles et al., 2005).

For cardiac and respiratory activities, WT reveals that the main frequency bands in which the power of these signal changes over time are within 0–45 and 0–12 Hz, respectively.

For all physiological variables, the power is color coded using the visible light spectrum.

For each segment of 30 s, we obtained the total power average from brain and cardiorespiratory activities in the following specific frequency bands: from brain (30–60 Hz) (Ramón et al., 2004; Mendoza-Ángeles et al., 2010), cardiac (0–2, 3–12, and 13–45 Hz), and respiratory (for each gill chamber: 0.5–3 and 3–12 Hz) electrical activity. Frequency bands for cardiac and respiratory activities were selected *via* WT analysis.

With these values, we constructed an ***n****-*by*-****p*** data matrix **x**. Rows of **x** correspond to the number of 30 s segments of the complete sleeping episode and columns correspond to each variable in which total power averages are calculated (considering the different frequency bands identified previously, eight variables make up the matrix **x**). For each complete sleeping episode, we obtained a specific matrix **x** with different number of rows and equal number of columns.

For each matrix **x**, we determined the temporal patterns of sleep over time by Pearson correlation matrices. Finally, to determine the relation between brain and cardiorespiratory electrical activity during sleep, we performed a *k-*means clustering to separate the segments of the ***n****-*by*-****p*** data matrix **x** into ***k*** clusters.

##### Pearson Correlation Matrix

The calculation of pairwise correlation coefficient on a dataset, known as the correlation matrix, is often used in data analysis, signal processing, pattern recognition, image processing and bioinformatics (Pratt, 1974; Brown, 1991; Gonzalez and Woods, 2002; Chang et al., 2009). Correlation matrix is a multi-variable analysis technique. By correlation matrix it is possible to study the time relationship (i.e., the evolution temporal of sleep), and the spatial relationship (relationship among variables). For our purpose, we used this technique to determine the temporal evolution of sleep for each matrix x.

##### *k*-means Clustering

The goal of cluster analysis was to discover the *natural* grouping(s) of this set of electrophysiological variables during the sleep state. The *k*-means algorithm is an unsupervised learning algorithm to cluster data based on their pairwise distances into *k* partitions, while minimizing overall intracluster variance.

The algorithm starts by partitioning the input points (*x*_j_) into *k* initial clusters, either at random or using some heuristic data. It then calculates the centroid of each set and computes the point-to-cluster-centroid distance of all points to each centroid to assign each *x*_j_ to the cluster with the closest centroid.

The new centroids are calculated for the new clusters. This process is iterated until convergence, which is obtained when none of the points switch to another cluster (Wittek, 2014).

For our cluster analysis, matrix **x** was normalized, so that the set of matrix elements has zero mean and unity variance. To perform the cluster algorithm, we used tidyverse and cluster libraries (Wickham et al., 2019; Maechler et al., 2021) in R software, for data manipulation and for methods of cluster analysis. Lastly, we used the NbClust library, which provides 30 indices for determining the number of clusters and proposes the best clustering scheme from the different results obtained by varying all combinations of number of clusters, distance measures, and clustering methods (Charrad et al., 2014).

We used PCA to plot data points according to the first two principal component coordinates.

##### Principal Component Analysis

Principal component analysis is a useful technique for exploratory data analysis, allowing us to better visualize the variation present in a dataset with many variables. It is particularly helpful in the case of “wide” datasets, where we have many variables for each sample (Abdi and Williams, 2010). In our analysis, we use PCA to reduce the number of dimensions of matrix **x** and outputs of two new variables (these represent the original variables maintaining as much variance as possible) that we use to do the plot of cluster results. We use the *fviz_cluster* function in the factoextra library in R software to extract and visualize the output of our multivariate data analyses.

#### Statistical Analysis

To estimate the probability that two samples of the results of our multivariate analysis stem from the same distribution, we employ the Kruskal–Wallis test (R software), to compare data before (1 min), during the complete sleeping episode, and after (1 min) the episode. The Wilcoxon test *post hoc* test was used. Differences were considered significant when *p* < 0.05. For the complete analysis, we estimated the epsilon squared (ε^2^) and effect size for Kruskal–Wallis to quantify possible discrepancies between clusters. We performed these analyses for each sleeping episode.

## Results

### How Does a Crayfish Sleep?

As previously reported (Ramón et al., 2004), the description of crayfish’s sleep was based on behavioral and electrophysiological criteria defined for vertebrates.

#### Behavioral Criteria

Crayfish behaving freely in an aquarium can be seen walking or lying on one side against the surface of water (Figure 1B), and it will stay in these positions for variable times regardless if it is daytime (Figure 1C). Lying on one side has been described as a stereotypical position for a sleeping crayfish, and it is accompanied by an increase in sensory threshold while a walking crayfish is associated with wakefulness (Figure 1B). However, continuous recordings from crayfish’s behavior show that it remains for considerable amounts of time motionless, with both chelae resting on the bottom of the aquarium and sometimes with antennae and antennulae lowered in a motionless (resting) position (Figure 1B). The analysis corresponding to each crayfish showed that the transition probabilities among waking, motionless, and lying on one side are 0.4 < *p* < 0.5; 0.3 < *p* < 0.4; and *p* ≈ 0.2, respectively. However, there are differences regarding the duration of each condition. Until now, the pattern of the brain’s electrical activity during this motionless position is unknown (see below).

#### Electrophysiological Criteria

The brain electrical activity from an alert animal (awake animals) is comprised by numerous spikes on a flat baseline; when crayfish is lying on one side (sleeping animals), these spikes are replaced by slow waves (see Figure 1D), as previously described (Ramón et al., 2004; Mendoza-Ángeles et al., 2010).

Recordings obtained from the crayfish’s brain were analyzed separately for 30 s segments using WT. Results are shown in Figure 2. We found that the brain electrical activity from sleeping animals shows clear differences for most frequencies from those of the awake animal. As previously reported (Ramón et al., 2004; Mendoza-Ángeles et al., 2010), the main change is a decrease in power for all frequencies, but the decrease is most pronounced for the frequency range of 30-45 Hz (high powers—red tones and low powers—blue tones), as compared to awake animals. However, our results show that decreases in power extend up to 60 Hz.

### Does Motionless Crayfish Sleep?

Continuous recordings from crayfish behaving freely in an aquarium show that the animal spends long periods of time motionless, as illustrated in Figure 1C. However, the brain’s electrical activity during this long-lasting period has still not been explored. Results of the corresponding WT-analysis are shown in Figure 3.

We found at least two different patterns when crayfish is immobile: 1) high power for all frequencies analyzed (0–60 Hz), like that of an alert (waking) crayfish (compare Figures 2A, 3A), and 2) a notable decrease in power mainly within the frequencies range of 30–60 Hz, which resembles that of an animal lying on one side (compare Figures 2B, 3B). We observed these two patterns in all segments from motionless crayfish analyzed.

### Sleep Phases in Crayfish

#### Brain Electrical Activity

Figure 4 represents the results of the WT analysis from a sleeping crayfish (lying on one side) for four non-consecutive 30 s windows. We found that during the same sleeping episode, the power of brain electrical activity is not constant but changes over time. Note that power decreases (Figure 4A) or increases (arrows in Figure 4B), particularly in the 30–60 Hz band, during different intervals. These increases are like those found in awake animals, which show high power in all analyzed frequencies (compare Figures 2A, 4B,C). These findings suggest that brain electrical activities during sleep show different patterns, which seems to indicate microstates. Such an apparent microstate is marked with arrows in segment **4B,** and segment **4C** represents another longer one. These microstates are present in different magnitudes and durations along the complete sleeping episode (Figure 5). Here we only considered those complete segments in which crayfish was sleeping while lying on one side.

Figure 5 shows the total average power over time of brain electrical activity within the 30-60 Hz frequency band (see Ramón et al., 2004; Mendoza-Ángeles et al., 2010), during a complete sleeping episode (segment 1 to segment 37), as well as 1 min before and 1 min after the sleep period. Each segment shows the probability distribution of the total average power during a 30 s period. Power is color coded using the visible light spectrum (high powers—light tones and low powers—dark tones). Before the sleeping episode, the total average power probability distribution shows a large tail, and the distribution is notably broader. At the beginning of the sleeping episode (segment 1), the widths of probability distribution decrease. As crayfish remains asleep, and the total average power decreases still further (after segment 7). Later, the average power and mean of probability distributions are maintained constant. However, occasionally some distributions fluctuate and are broader again, as before the sleeping episode (e.g., segments 19 and 25). Generally, the total average power, mean, and widths of the probability distributions increase after a sleeping episode. A Kruskal–Wallis test was used to compare data before (1 min), during the complete sleeping episode, and after (1 min) the episode. It showed significant differences among these groups with a p-value less than the significance level of 0.05. The Wilcoxon test *post hoc* showed that only the before and during the complete sleeping episode are significantly different (*p* < 0.05). The differences between during sleep and after sleep might be marginal, perhaps because more time is needed to wake the crayfish.

#### Cardiorespiratory Electrical Activity

To complete the picture of dynamical changes during crayfish’s sleep, we turn now to cardiac and respiratory electrical activity corresponding to waking and sleeping segments discussed in the previous section.

**FIGURE 6:**
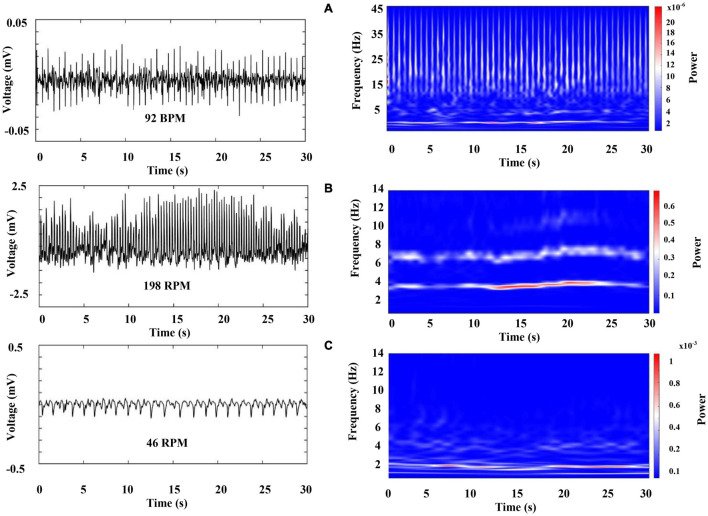
Representative raw recordings of cardiorespiratory electrical activity corresponding to an awake crayfish (left column) and color-coded representation of the time–frequency analysis by WT for these signals (right column). Each panel represents 30 s of activity. **(A)** Heart (electrocardiogram, ECG), **(B)** and **(C)** both gill chambers.

Figure 6 (left column) shows a characteristic raw recording of electrical activity from the (A) heart (electrocardiogram, ECG) (B) and (C) both gill chambers from a walking crayfish. The right column represents the results of a time–frequency analysis (WT) for these signals. Data for heart activity consist of slow components (1.5–12 Hz) punctuated by high-frequency transients (up to 45 Hz) that occur almost periodically. Our results show that during wakefulness, when the crayfish is active (walking), HR is around 100 BPM (beats per minute) and ECG amplitude changes slightly depending on the animal’s activity. We found that the spectral profile of crayfish’s ECG contains three main components: HR centered at very low frequencies (VLF) around 2 Hz, a low-frequency (LF) component located between 3 and 12 Hz, and high frequencies (HF) between 13 and 45 Hz.

**FIGURE 7:**
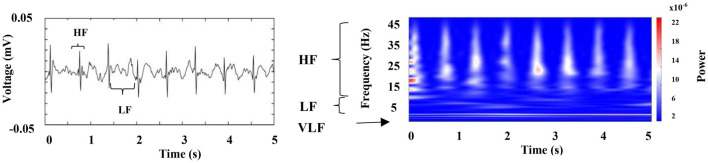
Representative raw recording of cardiac electrical activity corresponding to an awake crayfish (left panel); this graph indicates the main frequency components. Color-coded representation of the time–frequency analysis by WT for this activity (right panel); it points out the raw recording’s spectral profile. VLF (very low frequency), LF (low frequency), and HF (high frequency).

Figure 7 represents the first 5 s of the cardiac electrical activity shown in Figure 6A, visualizing the above mentioned frequency bands. We found that RF [respiratory frequency, respirations per minute (RPM)] ranges in two different frequency bands, (1) 0.5–3 Hz and (2) 3 to 12 Hz, and its central frequency can vary from 0.5 to 4.0 Hz. Respiratory signal’s power and amplitude are highly irregular, and there is no apparent synchronization between both chambers (compare Figures 6B,C, note the scale in the right column). We observed such patterns in all segments analyzed from the three crayfish.

**FIGURE 8:**
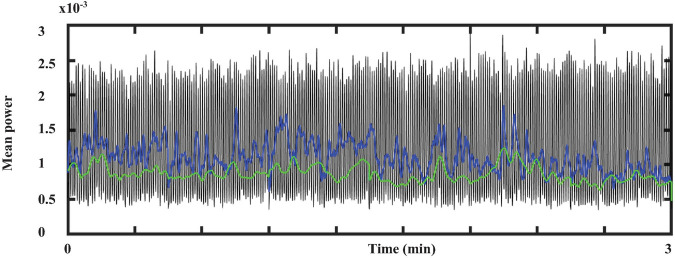
Total average power from ECG within the three-main-frequency band. Green line indicates the results obtained for band 1: 0.5–3 Hz, blue line those for band 2: 3–12 Hz; black line shows the total power average power for band 3: 13–45 Hz.

Figure 8 represents the total average power of ECG within the spectral decomposition previously mentioned. The Kruskal-Wallis test indicated that there is a significant difference (p-value < 0.05) among these frequency bands. Therefore, these results suggest that three main oscillatory components are present in ECG crayfish.

**FIGURE 9:**
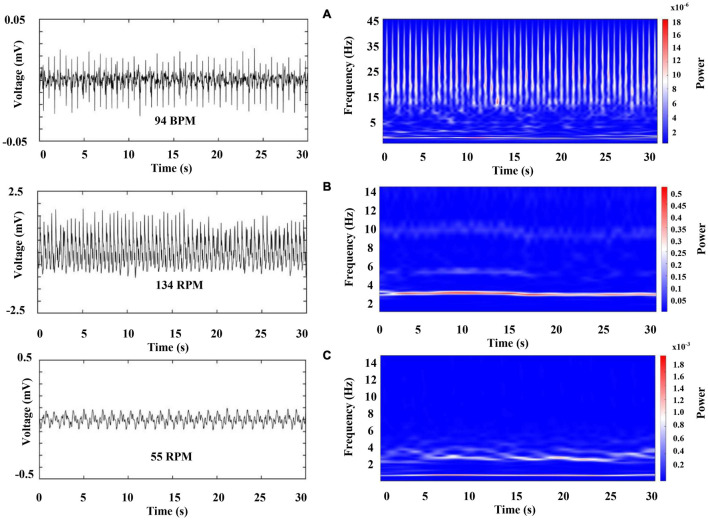
Representative recordings of cardiorespiratory electrical activity 2 min after the crayfish started to sleep. The left column shows raw data for **(A)** ECG (upper panel), **(B)** and **(C)** respiratory chambers (middle and low panel). The right column shows a color-coded representation of the time frequency analysis by WT for each signal. Each panel represents 30 s of activity.

**FIGURE 10:**
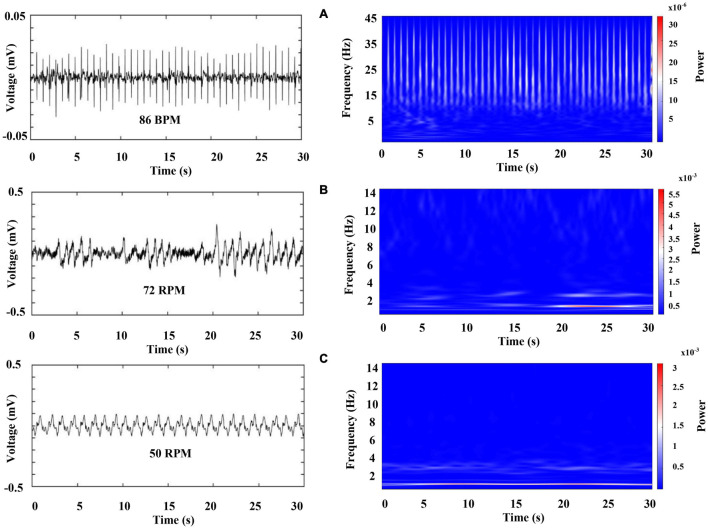
Representative recordings of cardiorespiratory electrical activity 10 min after sleep onset. The left column shows raw data for **(A)** ECG (upper panel), **(B)** and **(C)** respiratory chambers (middle and low panel). The right column shows the color-coded representation of time–frequency analysis by WT for each signal. Each panel represents 30 s of activity.

Next, we characterize the cardiorespiratory activity during sleep. Figure 9 was obtained 2 min after sleep onset (we marked a sleep onset once crayfish was clearly lying on one side without any sudden movements). Figure 10 belongs to the same sleep episode but 10 min after onset. Despite that the crayfish was lying on one side at the water surface, the respiratory data shown in Figure 9 resemble the findings of an awake animal with the similar subtle changes in power and frequency while the spectral profile of ECG shows a decrease in power (compare Figures 6, 9).

However, crayfish shows a progressive decrease in power of the VLF, LF, and HF components of ECG as well as in the two-frequency band identified in RF, as it is sleeping (compare Figures 9, 10, note the scale). These changes occur for a long sleeping episode and show differences and similarities with the pattern encountered during wakefulness.

**FIGURE 11:**
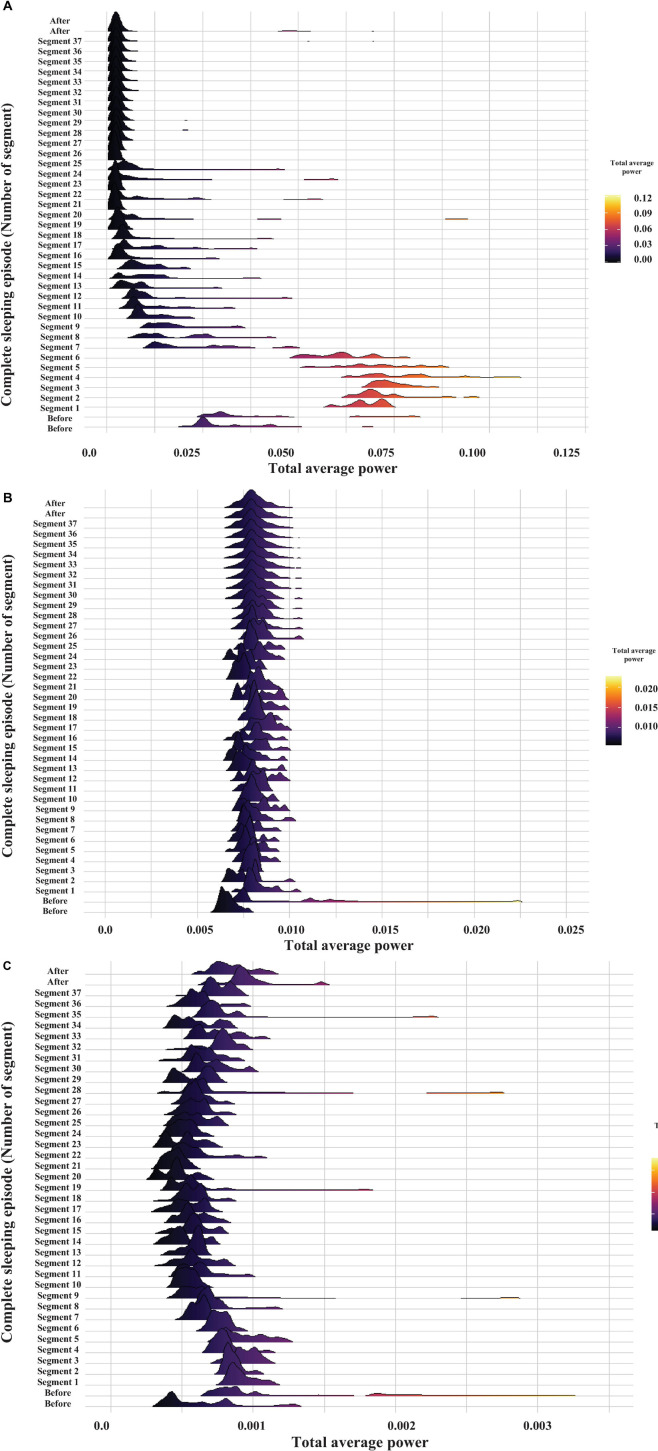
Probability distribution of cardiorespiratory electrical activity total average power within the following frequency bands: heart 0–45 Hz and respiratory 0–12 Hz signal. The graph displays the time evolution of the probability distribution over a complete sleeping episode of about 20 min from bottom to top. Each segment shows the probability distribution of total average power during the 30 s windows. The beginning of the sleeping episode is marked with segment number 1, and it finishes at segment number 37. The figure also includes 1 min before and one after the sleeping episode. The power values were obtained by the WT analysis and are coded in color (high powers—light tones and low powers—dark tones). **(A)** gill chamber1, **(B)** gill chamber2, and **(C)** heart.

Figure 11 shows the behavior of cardiorespiratory electrical activity during a long-lasting sleeping episode of a duration of about 20 continuous minutes. We also include a minute before and after the sleeping episode. We used the total average power in the frequency bands described above (heart 0–45 Hz and respiratory 0–12 Hz signal) to build these graphs.

In general, we found that before the sleeping episode and during its beginning the cardiorespiratory activity’s total average power shows broader distributions during the initial part and a notable decrease of the average power combined with more narrow distributions toward the final part of the episode. The total average power appears to decrease as a function time. Note how the average power decreases still further while crayfish remains asleep (Figure 11, compare the patterns at the beginning of the sleeping episode (segment 1) and after segment 7. Note the scale). Later, the average power and mean of probability distributions are maintained constant. However, occasionally some distributions fluctuate and are broader again, as before the sleeping episode.

The probability distribution of brain electrical activity shown in Figure 5 corresponds to the same complete sleeping episode shown in Figure 11. Note how this general pattern in cardiorespiratory electrical activity is also present in brain electrical activity. These transitions, between a phase, with low heart and respiratory activity, and a next one that resembles an active animal despite that it was lying on one side at the surface of the water, repeated mainly when the animal lasted long periods of sleep. Although the average power decreases too, shorter complete sleeping episodes show probability distributions with large tails and distributions notably broader over time, both for the total average power of brain and for cardiorespiratory electrical activity (see Supplementary Materials to revise more sleeping episodes). Interestingly, we found a different pattern between both gill chambers during wakefulness and sleep. This apparent desynchronization between gill chambers was found in all segments analyzed (e.g., compare panel B and C in Figures 6, 9, 10) from the three crayfish. To determine if there is any significant difference between the data before (1 min), during the complete sleeping episode, and after (1 min) the episode, we compute a Kruskal–Wallis test. It showed significant differences between these data (*p* < 0.05). Comparison between before and sleep periods shows statistical differences. We also found differences between most of the sleeping periods and 1 min after (*p* < 0.05).

#### Brain and Cardiorespiratory Electrical Activity

We determined the temporal evolution of sleep considering the power average values from the brain (30–60 Hz), cardiac (all frequency bands), and respiratory (all frequency bands) into segments of 30 s. To this end, we constructed a Pearson correlation matrix for each complete sleeping episode (at least 25 episodes were analyzed). This matrix allows us to compare time and spatial relationships among multiple variables. Therefore, we divided each sleeping episode in 30 s segments and obtained the total power average for each segment from the brain, and cardiorespiratory activities in the following frequency bands: brain, 30–60 Hz, cardiac, 0–2, 3–12, and 13–45 Hz. Finally, for respiratory activity the bands were (for each gill chamber) 0.5–3 and 3–12 Hz. The obtained values allowed us to construct an ***n***-by-***p*** matrix. Rows correspond to each variable (eight variables), and columns correspond to each 30 s segment of the complete sleeping episode.

**FIGURE 12:**
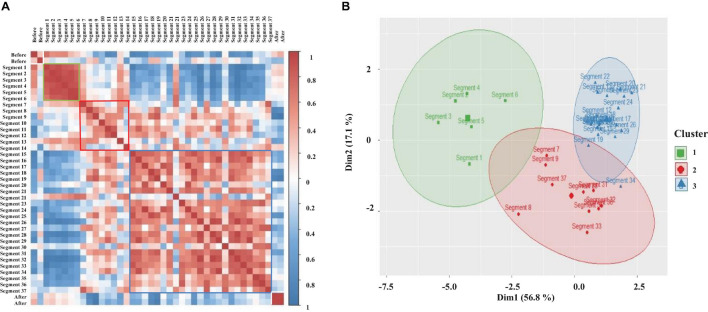
**(A)** Pearson correlation matrix derived from brain and cardiorespiratory electrical activity total average power. This displays the temporal evolution of sleep over a complete sleeping episode of about 20 min. Each segment shows the coefficient correlation of total average power into 30 s windows. The beginning of the sleeping episode is marked with segment number 1, and it finishes at segment number 37. The figure also includes a minute before and after sleeping episode (white square). The correlation is coded in color (positive correlations—dark red and negative correlations—dark blue. Each qualitatively identified cluster is framed with colors square: cluster 1 (green square), cluster 2 (red square), cluster 3 (blue square). **(B)** *k-*means clustering corresponding to the same sleeping episode shown in A; each cluster represents a different pattern during sleep. The observations of data are represented by points using principal components, PCA1 explains 56.8% of total variance and PCA2 explains 17.1%. A concentration ellipse was drawn around each cluster. Green square—cluster 1, red diamonds—cluster 2, and blue triangles—cluster 3.

Results for the correlation matrix corresponding to the complete sleeping episode shown in Figures 5, 11 are presented in Figure 12A. It also includes the correlation before (1 min) and after (1 min) the sleeping episode. Correlation is coded in color (positive correlation—red tones and negative correlation—blue tones). All diagonal elements are shadowed dark-red and represent the maximum correlation, 1. As expected from the individual analysis, the correlation matrix clearly differentiates among before, during the sleeping episode (segment 1 to segment 37), and after the sleeping episode. In the same way, it is possible to observe how during a complete sleeping episode a temporal interrelation exists. Note how the correlation matrix identifies at least three different patterns when crayfish is sleeping. Each pattern is framed with colored squares into qualitatively identified clusters. A well-defined cluster (green square) with high values for correlation and composed by the first segments (segment 1 to segment 6) is followed by a second group (red square) with a correlation pattern highly unstable and, finally, a third cluster (blue square) less homogeneous than the first one is present at the end of the episode. Note how the second cluster connects clusters 1 and 3, and its correlation pattern appears to be comparable to that of the third cluster. In general, the correlation pattern presented at the beginning of sleep changes over time; it suggests that the characteristics and patterns of sleep derived from brain and cardiorespiratory electrical activity total average power change over time, too. Once we found that a temporal interrelation exists during sleep, we analyzed each complete sleeping episode by *k-*means clustering to get a clear measure about the number of the clusters present. For this purpose, only the data corresponding to sleep episode were considered. To avoid any bias, we computed 30 indices for determining the number of clusters (see Data Analysis). Results for clustering analysis corresponding to the same complete sleeping episode are presented in Figure 12B.

*k*-means Clustering appears to have clustered the different pattern derived from the total power average data from sleeping crayfish’s brain and cardiorespiratory electrical activity into three distinct clusters. The observations (see Data Analysis section) are represented by points using principal components, and we drew a concentration ellipse around each cluster. The first cluster is composed of a few segments (green ellipse), these values corresponding with the beginning of the sleeping episode (around 3 min), and it is the smallest cluster. The second cluster is represented with a red ellipse; this is mainly related to probability distributions broader with large tails. The last one (blue ellipse) concentrates most observations. These results are consistent with the pattern shown in the correlation matrix (compare Figures 12A,B). To determine if there is any significant difference between these three groups, we used a Kruskal–Wallis test; by using the Wilcoxon test *post hoc*, we determined which pairs of groups were different, and epsilon squared (ε^2^) was calculated to quantify the size of the difference between clusters. The results show statistically significant differences among the three clusters with medium effect size (*p* < 0.05; ε^2^ = 0.25). However, the *post hoc* test shows mainly clear significant differences between the first group and the last group (*p* < 0.05). This strongly suggests that the second group may share characteristics with the other two groups, and it maybe a phase of transition in which patterns from group 1 and 3 coexist. For each sleep episode (see Supplementary Material to review more sleep episodes), we estimate the same measurements, and the results are consistent.

#### Sleep Phases in Crayfish: Case-Specific or Generic?

**FIGURE 13:**
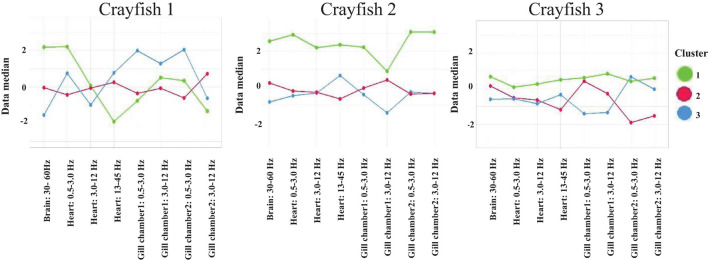
*k-*means clustering at the group level derived from brain and cardiorespiratory electrical activity total average power. Each panel represents the global clustering per crayfish; each one includes all corresponding sleeping episodes. For each cluster, the median for the corresponding variable is represented. Cluster 1 (green line), cluster 2 (red line), and cluster 3 (blue line).

Until now, we have demonstrated that in each complete sleeping episode a correlation pattern exits; at least three different patterns were identified (each one is represented by a cluster). We have presented detailed results for a sleeping episode. It remains to be shown whether these patterns reflect a crayfish-specific characteristic of brain and cardiorespiratory electrical activity or whether these represent more general features or a generic pattern across crayfish. For this purpose, we realized a *k*-means clustering at the group level, which included all corresponding sleeping episodes per crayfish. Results of this analysis are shown in Figure 13.

The analysis at the group level derived from brain (30-60 Hz), cardiac (all frequency bands), and respiratory (all frequency bands) electrical activity per crayfish shows similarities between crayfish sleep. Note how each crayfish presents three different clusters during sleep (into each cluster the data median corresponding with each variable is represented). Cluster 1 (green line) has no clear trend, although it seemingly presents higher values than the other groups. Clusters 2 and 3 (red and blue line) show a pattern seemingly anti-correlated between them, although they appear to share some characteristics. Each cluster presents specific characteristics derived from brain and cardiorespiratory activity. Note how these three groups identified during sleep are presented in the three crayfish studied. This strongly suggests that sleep’s general patterns are conserved among crayfish. For each crayfish, the Kruskal–Wallis test showed significant differences between the three groups found (*p* < 0.05). By using the Wilcoxon test *post hoc*, we found clear significant differences among these groups with medium effect size (*p* < 0.05; ε^2^ > 0.23). However, as is observed in Figure 13, in the case of crayfish 1, the differences between cluster 1 and cluster 2 are marginal; this makes sense because *k*-means shows that cluster 2 preserves characteristics common to the rest of the groups.

## Discussion

In general, our results are consistent with the previous description about sleep in crayfish (Ramón et al., 2004; Mendoza-Ángeles et al., 2007, 2010). However, we found that this large decrease in power within the 30-45 Hz band extends up to 60 Hz (Figures 2, 3). These results suggest that it remains to be explored if the pronounced decrease in signal power extends to frequencies above 60 Hz. Furthermore, the physiological relevance for such behavior is completely unclear but worth investigating.

According with our behavioral analysis (Figure 1C), crayfish shows a third position in which it remains motionless for a considerable amount of time with both chelae resting on the bottom of the aquarium and sometimes with antennae and antennulae lowered and motionless (resting). During this time, we found *via* WT analysis that the brain electrical activity shows characteristics in between that lying on one side and that waking (Figures 2, 3). In many cases, we found the same decrease in power at frequencies 30-60 Hz; in others, values were closer to those from awake animals. Therefore, these results possibly also indicate another sleeping state in crayfish.

### Sleep Phases in Crayfish

The wavelet analysis of crayfish’s EEG power during a long-lasting sleeping episode showed that slow-wave sleep in crayfish is not a continuous state (Figures 4, 5). The depth of sleep, measured as the power of EEG activity, changes over time, and power decreases while sleep deepens (see Figure 5); this strongly suggests that crayfish has sleep phases. The study from the brain in conjunction with the cardiorespiratory activity allows us to determine that sleep in crayfish is comprised by phases of various durations that do not seem to have a cyclic pattern (Figures 5, 11, 12). Our results suggest that crayfish has at least three different sleep phases: phase (1) drowsy period: at the beginning of the sleep period, the EEG and cardiorespiratory power show high values, and crayfish does not fall asleep immediately after lying on one side; like vertebrates, it requires some time to fall asleep. Generally, this pattern changes after about 3 min (Figures 5, 11, 12, segment 6) and eventually, it fades out to phase (2) phase transition: this represents moderate deep sleep. After-sleep-onset wave power decreases with time (segments 7 to 14, Figures 5, 11, 12). Phase (3) is the deepest level of sleep: it is characterized by a further decrease in EEG and cardiorespiratory activity power, together with short, intermittent bursts of high-power waves (segments 9 to 37, Figures 5, 11). We found that each one of these phases is conserved across crayfish (Figure 13). Although these results suggest that crayfish present sleep phases as in vertebrates, it is important to emphasize that they are quite different, and we do not intend to equate both and do not suggest similar generating mechanisms. A possible reason for the difference between sleep and sleep phases in crayfish and vertebrate animals is because the brain structure and the architecture are completely different. In this invertebrate, there are no cortices or cortex-like structures, nuclei, or an organization that resembles the vertebrate brain. Despite the major anatomical, physiological, and pharmacological differences between vertebrates and invertebrates, and the relatively small number of neurons in crayfish’s brain, this species shows complex behaviors like sleep. Our study demonstrates, behaviorally and electrophysiologically, that sleep in crayfish, as in mammals and birds, is a dynamic and heterogeneous state. This suggests that sleep is a conserved function and the different sleep phases are a fundamental characteristic of sleep, maybe in any animal.

### Physiological Changes During Sleep Phases in Crayfish

In mammals, sleep stages are well characterized, and it is known that a variety of additional physiological changes take place during the different sleep stages as compared to wakefulness. In vertebrates, these changes are mediated by the autonomic nervous system (ANS), whose actions are mediated by the sympathetic nervous system and the parasympathetic nervous system.

The ANS activity changes during sleep; HR, blood pressure, and respiratory rate diminish. During REM sleep, HR increases again showing a high variability which may exceed that observed during quiet wakefulness (Zemaityte et al., 1986).

The panorama seems quite different for invertebrates, particularly crustaceans. In this group of animals, there are no anatomical structures resembling an ANS, but there are behavioral and cardiorespiratory responses indicative of an autonomic-like regulation. Social interactions or environmental changes induce modifications in HR and RF (Schapker et al., 2002; Shuranova et al., 2006; Cooper et al., 2011; Canero and Hermitte, 2014). Recently, we reported for the first time that changes in these variables occur during sleep, too (Osorio-Palacios et al., 2021). Here, we studied if these physiological changes take place during the different sleep stages.

Our results show that in crayfish, the heart rate and respiratory frequency are regulated during wakefulness (Figures 6, 7) and during the different sleep phases (Figure 11), as it occurs in vertebrates. By using the Pearson correlation matrix and *k-*means clustering, we found that brain and cardiorespiratory activity are related during sleep. The last one confirmed that data can be arranged in three clearly separated groups (Figures 12B, 13), which gives support to the idea of three completely different sleep phases determined for the brain in conjunction with cardiorespiratory activity. According to these, these phases of sleep are accompanied by changes in autonomic variables.

Electrocardiogram (ECG) frequency variations have been studied extensively in vertebrates. Three main oscillatory components are present in HR vertebrates variability, very low frequency (VLF), marker of hormonal and circadian oscillations, low-frequency component (LF), marker of sympathetic modulation, and high-frequency component (HF), marker of vagal modulation and synchronous with respiration (Montano et al., 2009). Electrocardiogram analysis has been widely used for the assessment of cardiovascular autonomic control during sleep, showing a progressive decrease of the LF component, marker of sympathetic modulation, and a predominant vagal control, as sleep becomes deeper (from wakefulness to deep NREM sleep). Rapid eye movement sleep is characterized by a predominant sympathetic modulation with surges of sympathetic activity at levels even higher than in wake conditions (Trinder et al., 2001; Brandenberger et al., 2003; Legramante et al., 2003; Tobaldini et al., 2014).

In the case of crayfish, so far we identified three main oscillatory components from ECG signals (VLF around 2 Hz, LF 3 to 12 Hz, and HF between 13 and 45 Hz) (Figure 10), and these results were enough to achieve our main goal, but they suggest that a detailed study from cardiac variability in crayfish would provide more evidence about the existence of a functional ANS as it happens in vertebrates.

As we previously mentioned, in crayfish there are no descriptions of an ANS, and we ignore the mechanisms and pathways mediating this regulation of cardiorespiratory activity during wakefulness and sleep. One possibility is that excitatory (sympathetic-like) and parasympathetic-like circuits would be allocated in a region of brain named tritocerebrum. Field potential oscillations have been described previously in this brain area (Ramón et al., 2004; Arellano-Tirado, 2016), and they are concomitant with heartbeat. Another possibility relays in the suboesophageal ganglion, where command neurons were reported a long time ago (Wiersma and Novitski, 1942; Maynard, 1960; Taylor, 1970; Field and Larimer, 1975a,b). We do not have evidence of an equivalent mechanism for respiratory regulation. Therefore, we can postulate at least two circuits allocated in the tritocerebrum and/or the suboesophageal ganglion that have excitatory (sympathetic-like) and inhibitory (parasympathetic-like) activity, regulate both cardiovascular and respiratory activities, and receive strong input from the deutocerebrum and protocerebrum during wake conditions; this input would diminish during sleep, and that would be the reason for heart and respiratory activity diminution and dispersion.

## Conclusion

In this study, we analyzed physiological time series from crayfish brain and cardiorespiratory electrical activity by WT, Pearson correlation matrix, and unsupervised learning techniques (*k-*means analysis) to search for sleep phases and determine the relationship between these activities during sleep. These techniques allow us to state that (1) in crayfish there are at least three different sleep phases and (2) changes in physiological variables like HR and RF take place during different sleep phases. Sleep phase 1 (drowsy period): EEG has high power and is accompanied by cardiorespiratory electrical activity also of high power, as high as those found in wakefulness. This phase is present at the beginning of sleep. Sleep phase 2 (transition phase) presents a further decrease in power of EEG waves and cardiorespiratory activity; this phase can present characteristics from phases 1 and 3. Sleep phase 3 (deepest level of sleep): the predominant EEG power consists of low power, with some burst of high-power activity; cardiac frequency and respiratory frequency are also reduced to their lowest values during this phase. (3) We defined the depth of sleep in accordance with the reduction in the wave power for EEG and the concomitant reductions in both cardiac and respiratory frequencies. (4) Finally, we propose that sympathetic-like and parasympathetic-like circuits lay down in tritocerebrum and/or the suboesophageal ganglion; they regulate cardiac and respiratory activities (at least) and receive modulatory inputs from the upper regions of the brain.

All these conclusions support the view that crayfish’s characteristics from an evolutionary perspective would be useful to find out how this behavior originated and evolved to reach the complexity seen in vertebrates. The main purpose in future studies will be to characterize the different sleep phases.

## Data Availability Statement

The raw data supporting the conclusions of this article will be made available by the authors, without undue reservation.

## Author Contributions

MO-P, KM-Á, and JH-F designed the research and the experiments for the data analysis. MO-P and LM-T collected the data. MO-P, KM-Á, and IO-D wrote the code, analyzed the data, and produced the figures. All authors participated in the discussion of the results, reviewed, and approved the final version of the manuscript.

## Conflict of Interest

The authors declare that the research was conducted in the absence of any commercial or financial relationships that could be construed as a potential conflict of interest.

## Publisher’s Note

All claims expressed in this article are solely those of the authors and do not necessarily represent those of their affiliated organizations, or those of the publisher, the editors and the reviewers. Any product that may be evaluated in this article, or claim that may be made by its manufacturer, is not guaranteed or endorsed by the publisher.
