# Supplementary material for: Sleep Phases in Crayfish: Relationship Between Brain Electrical Activity and Autonomic Variables
Source: Front Neurosci. 2021 Oct 14;15:694924. doi: 10.3389/fnins.2021.694924 (PMC8551808; doi:10.3389/fnins.2021.694924)
Supplement: Supplementary file 1 [file Data_Sheet_1.PDF]

Complete sleeping episode 1 – crayfish 1

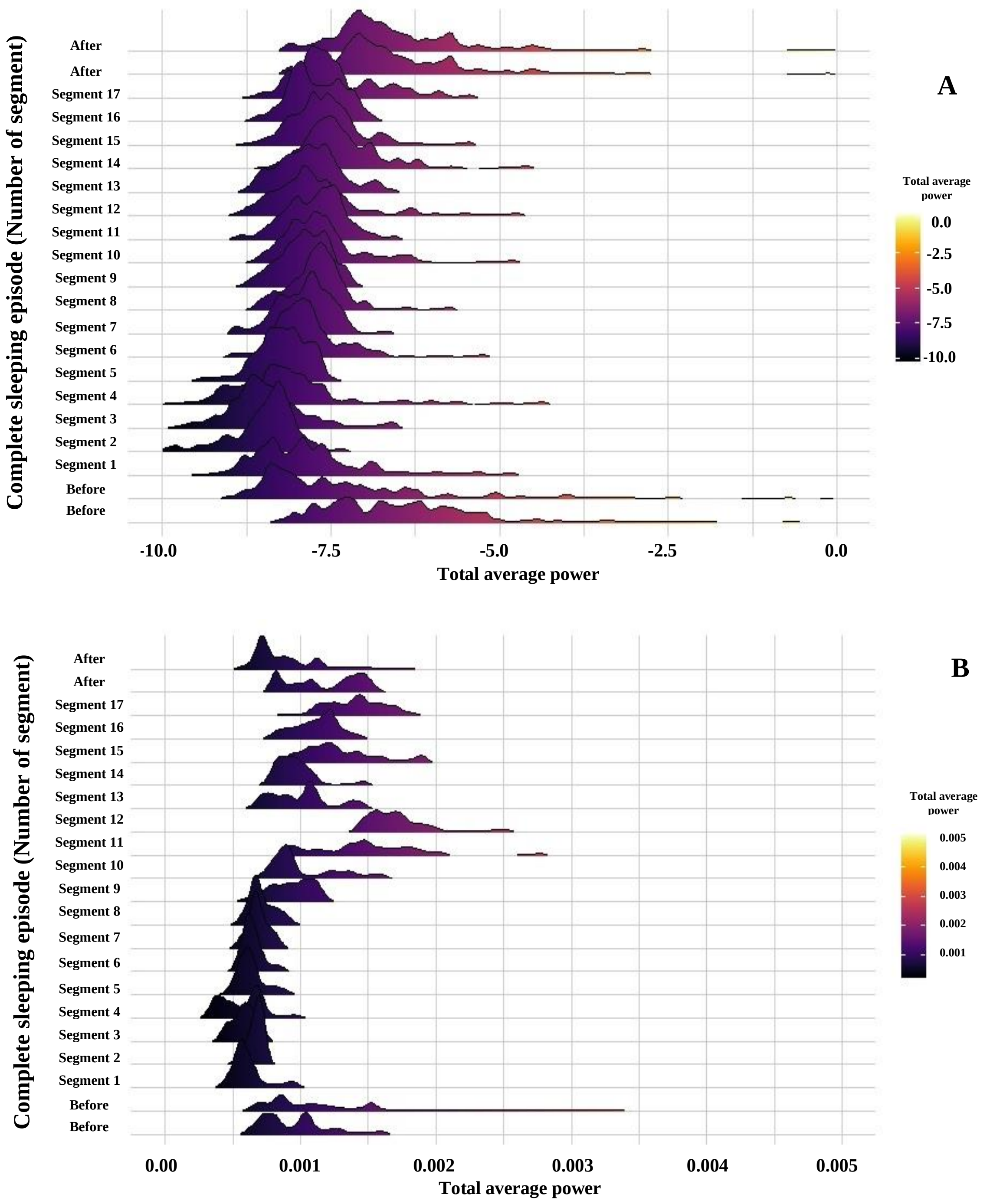

Supplementary. Probability distribution of the total average power of brain and cardiac electrical activity within the frequency band of 30-60 Hz and 0-45 Hz, respectively. The graph displays the time evolution of the probability distribution over a complete sleeping episode of about 8.5 minutes from bottom to top. Each segment shows the probability distribution of total average power during 30 second windows. The beginning of the sleeping episode corresponds to segment number 1, and it finishes at segment number 17. The figure also includes a minute before and one after the sleeping episode. The power values were obtained by the WT analysis and are coded in color (high powers-light tones and low powers-dark tones). **(A)** brain, **(B)** heart.

Complete sleeping episode 1 – crayfish 1

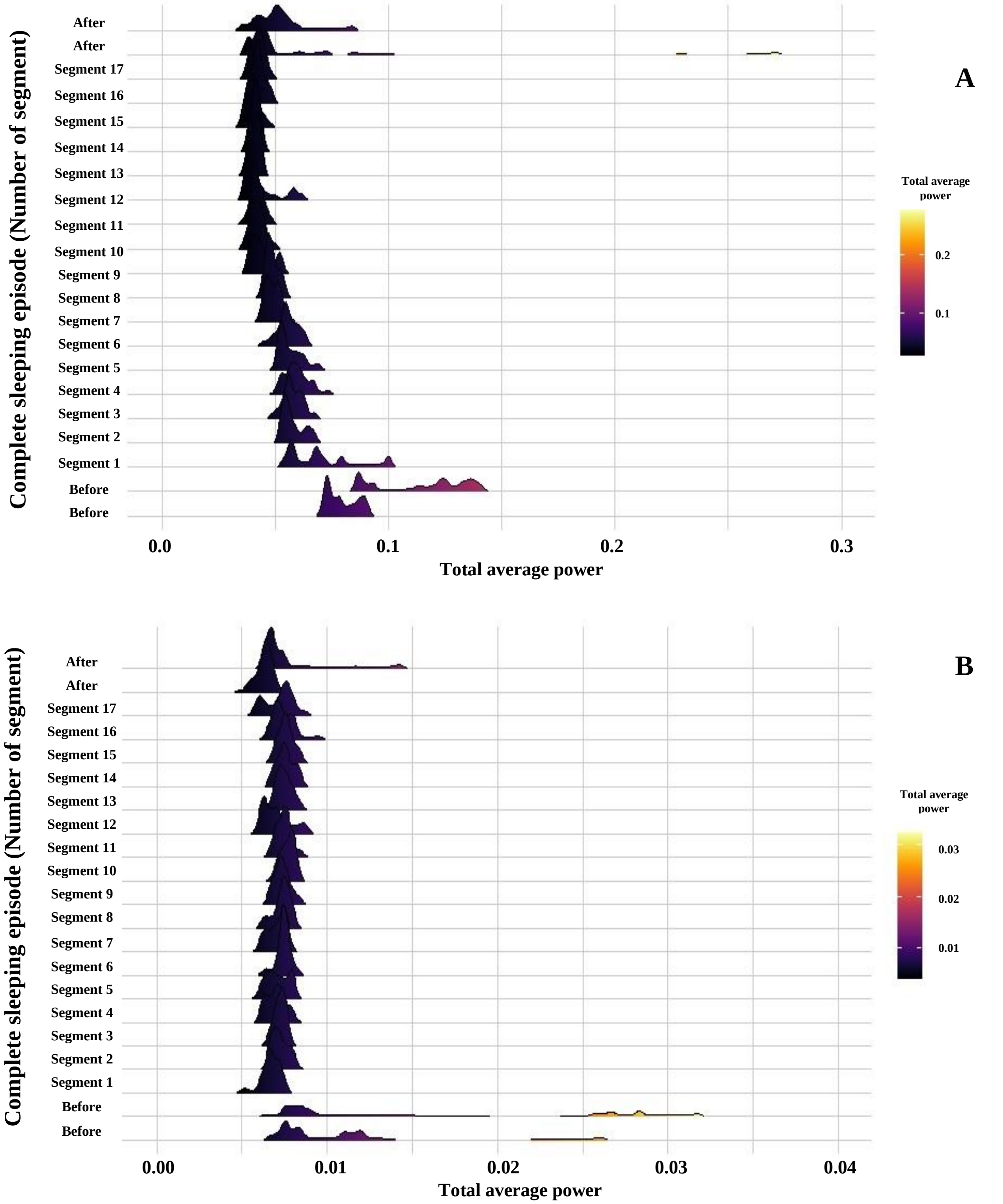

Supplementary. Probability distribution of the total average power of respiratory electrical activity within the frequency band of 0-12 Hz. The graph displays the time evolution of the probability distribution over a complete sleeping episode of about 8.5 minutes from bottom to top. Each segment shows the probability distribution of total average power during 30 second windows. The beginning of sleeping episode corresponds to segment number 1, and it finishes at segment number 17. The figure also includes one minute before and one after the sleeping episode. The power values were obtained by the WT analysis and are coded in color (high powers-light tones and low powers-dark tones). **(A)** gill chamber 1, **(B)** gill chamber 2.

Complete sleeping episode 1 – crayfish 1

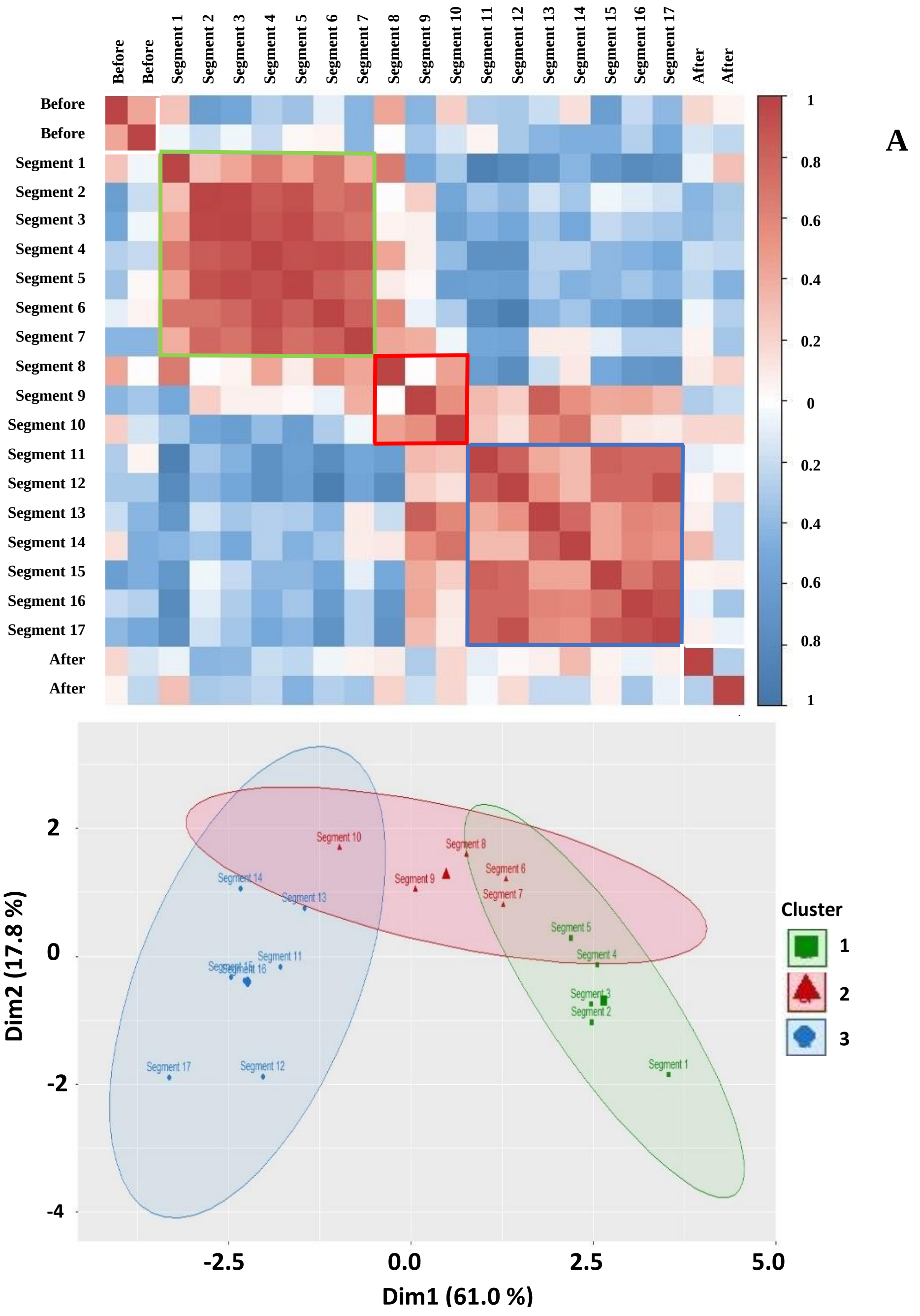

Supplementary. (A) Pearson correlation matrix derived from brain and cardiorespiratory electrical activity total average power. This displays the temporal evolution of sleep over a complete sleeping episode of about 8.5 minutes. Each segment shows the coefficients correlation of total average power in 30 seconds windows. The beginning of the sleeping episode is marked with segment number 1, and it finishes at segment number 17. The Figure also includes one minute before and one after the sleeping episode (white square). The correlation is coded in color (positive correlations-dark red and negative correlations-dark blue. Each qualitatively identified cluster is framed with a colored square: green square (cluster 1), red square (cluster 2), blue square (cluster 3). **(B)**. *k*-Means clustering corresponding to the same sleeping episode showed in A, each cluster represent a different pattern during sleep. The observations of data are represented by points using principal components, PCA1 explains 61.8 % of total variance and PCA2 explain 17.8%. A concentration ellipse was drawn around each cluster. Green squares (cluster 1), red triangles (cluster 2) and blue diamonds (cluster3). Results shows statistically significant differences between the three clusters with medium effect size ($p < 0.05$; $\varepsilon^2 = 0.15$).

Complete sleeping episode 2 – crayfish 2

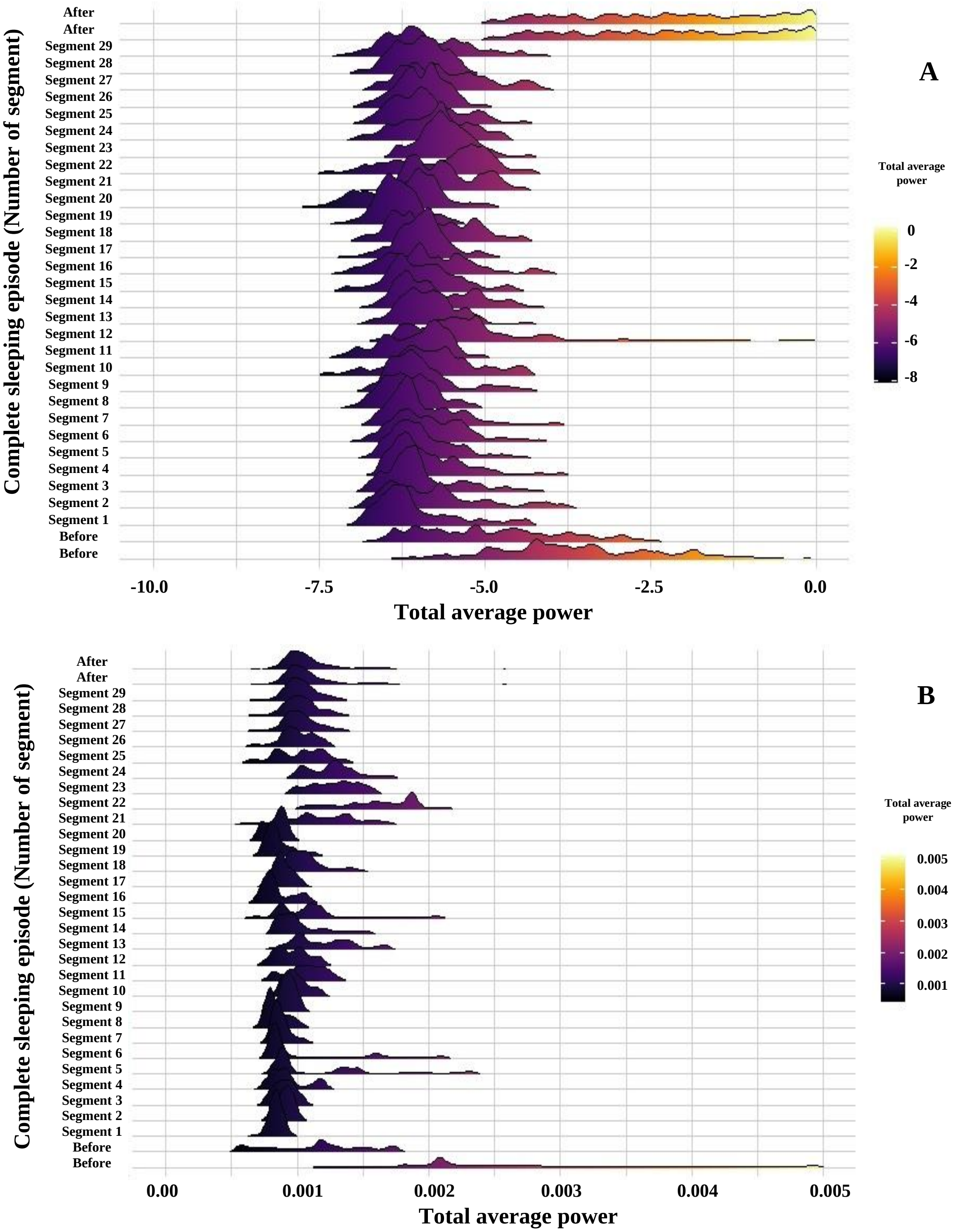

Supplementary. Probability distribution of the total average power of brain and cardiac electrical activity within the frequency band of 30-60 Hz and 0-45 Hz, respectively. The graph displays the time evolution of the probability distribution over a complete sleeping episode of about 14.5 minutes from bottom to top. Each segment shows the probability distribution of total average power during 30 second windows. The beginning of the sleeping episode corresponds to segment number 1, and it finishes at segment number 29. The figure also includes one minute before and one minute after the sleeping episode. The power values were obtained by the WT analysis and are coded in color (high powers-light tones and low powers-dark tones). **(A)** brain, **(B)** heart.

Complete sleeping episode 2 – crayfish 2

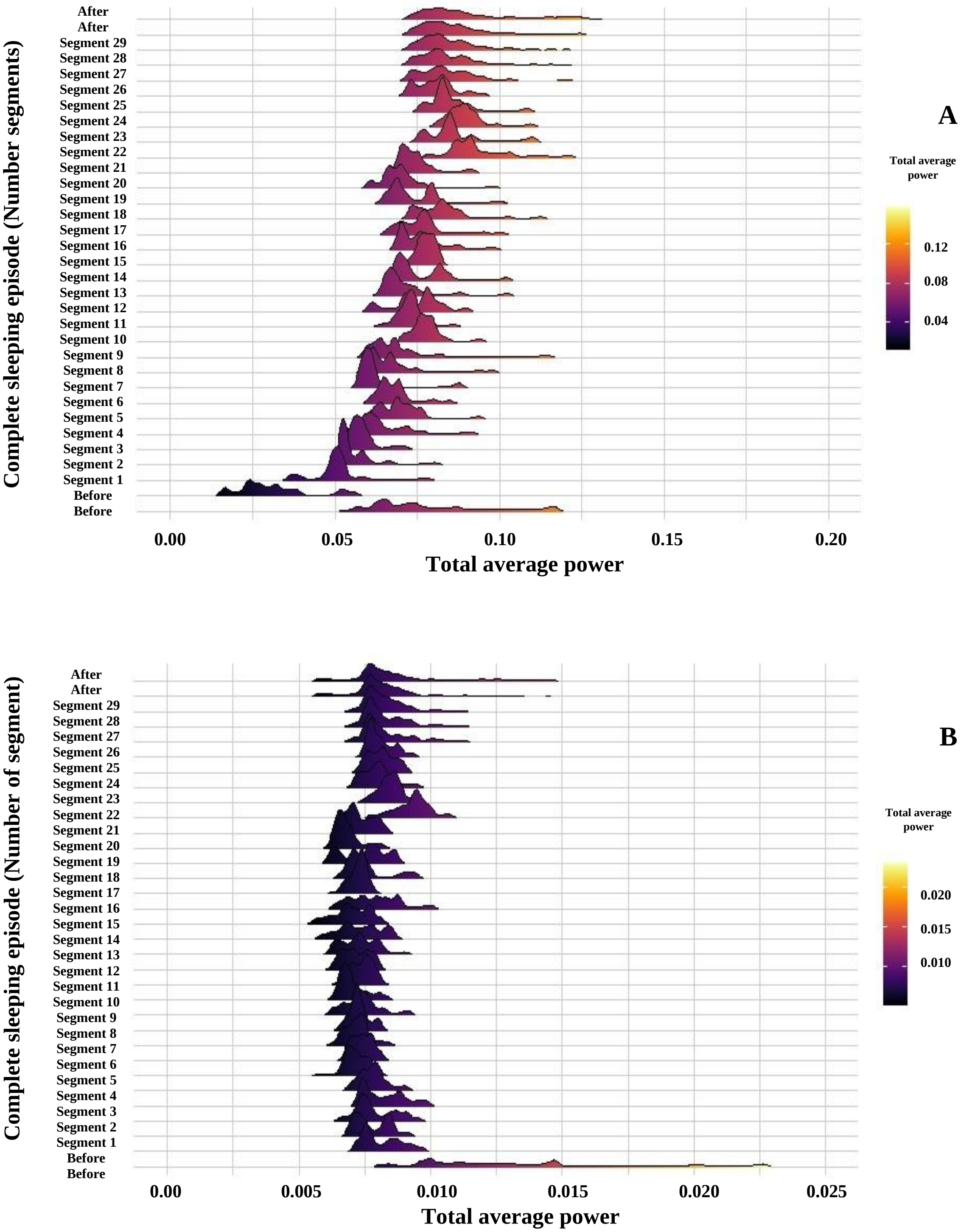

Supplementary. Probability distribution of the total average power of respiratory electrical activity within the frequency band of 0-12 Hz. The graph displays the time evolution of the probability distribution over a complete sleeping episode of about 14.5 minutes from bottom to top. Each segment shows the probability distribution of total average power during 30 seconds windows. The beginning of the sleeping episode corresponds to segment number 1, and it finishes at segment number 29. The figure also includes one minute before and one after the sleeping episode. The power values were obtained by the WT analysis and are coded in color (high powers-light tones and low powers-dark tones). **(A)** gill chamber 1, **(B)** gill chamber 2.

Complete sleeping episode 2 – crayfish 2

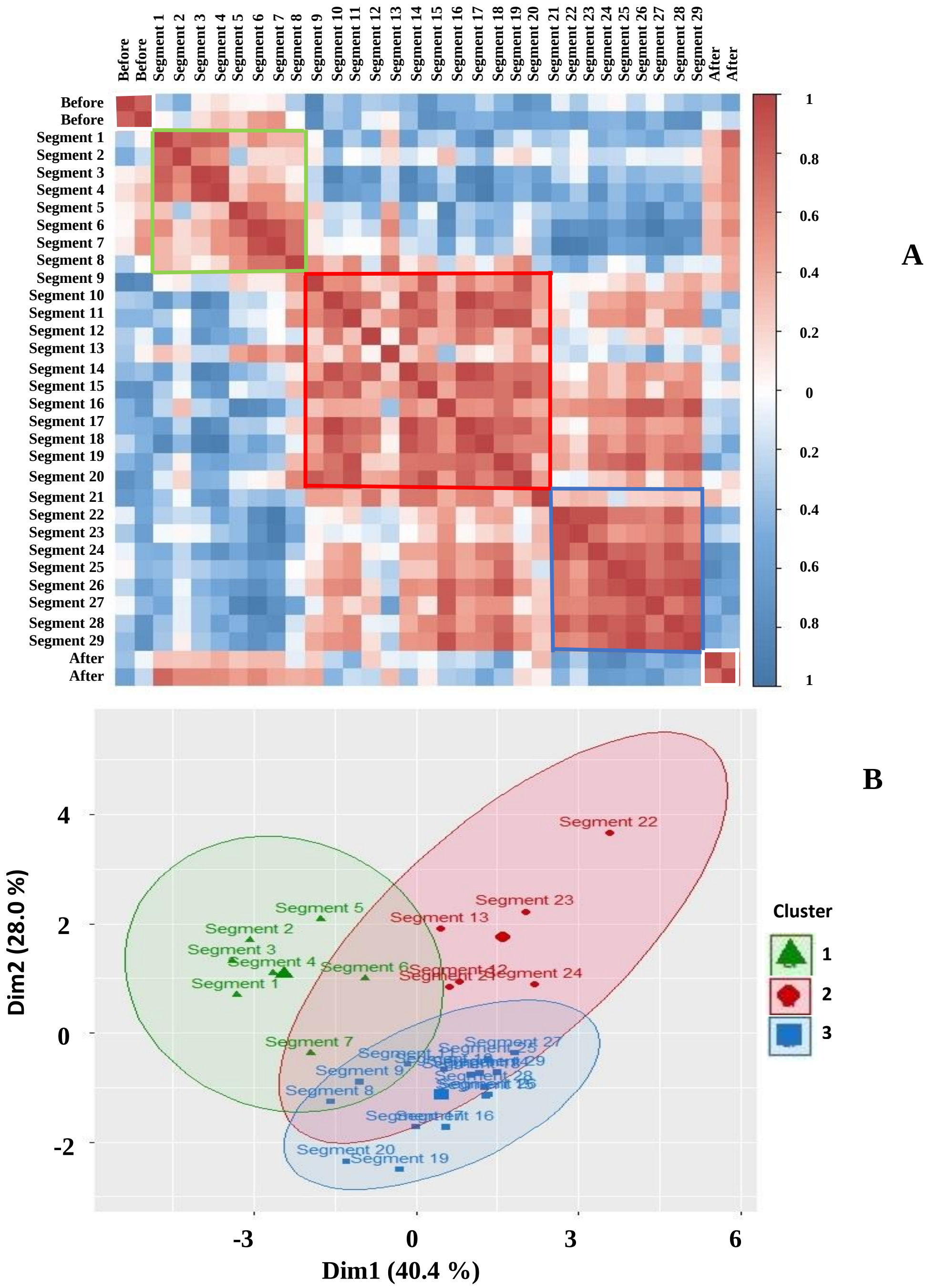

Supplementary. (A) Pearson correlation matrix derived from brain and cardiorespiratory electrical activity total average power. This displays the temporal evolution of sleep over a complete sleeping episode of about 8.5 minutes. Each segment shows the coefficients correlation of total average power into 30 seconds windows. The beginning of the sleeping episode is marked with segment number 1, and it finishes at segment number 29. The Figure also includes one minute before and one minute after the sleeping episode (white square). The correlation is coded in color (positive correlations-dark red and negative correlations-dark blue. Each qualitatively identified cluster is framed with a colored square: green square (cluster 1), red square (cluster 2), blue square (cluster 3). **(B)**. *k*-Means clustering corresponding to the same sleeping episode showed in A, each cluster represent a different pattern during sleep. The observations of data are represented by points using principal components, PCA1 explains 40.4 % of total variance and PCA2 explain 28.0 %. A concentration ellipse was drawn around each cluster. Green triangles (cluster 1), red diamonds (cluster 2), and blue squares (cluster3). Results shows statistically significant differences among the three clusters with medium effect size ($p < 0.05$; $\varepsilon^2 = 0.15$).

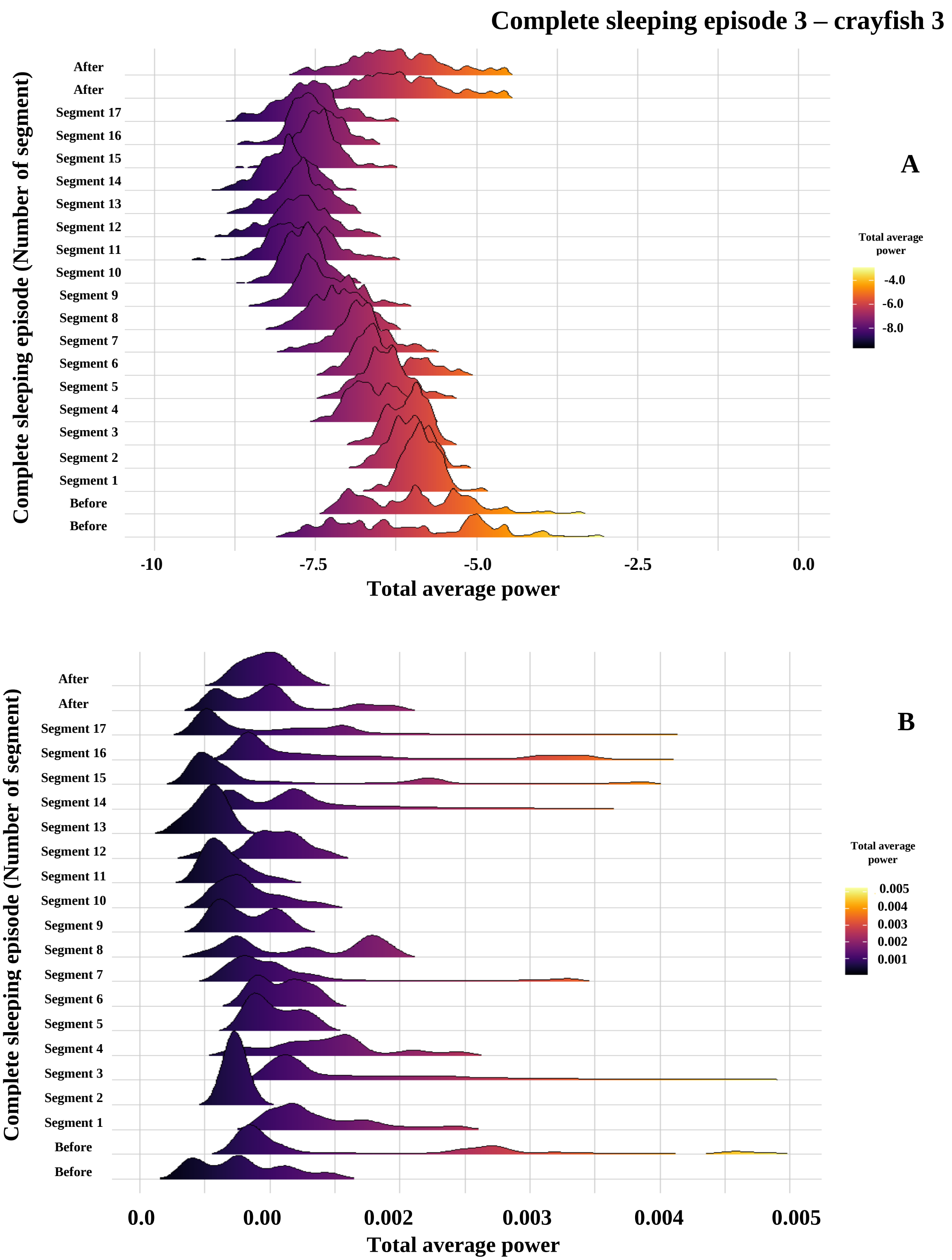

Supplementary. Probability distribution of the total average power of brain and cardiac electrical activity within the frequency band of 30-60 Hz and 0-45 Hz, respectively. The graph displays the time evolution of the probability distribution over a complete sleeping episode of about 8.5 minutes from bottom to top. Each segment shows the probability distribution of total average power for 30 seconds windows. The beginning of the sleeping episode corresponds to segment number 1, and it finishes at segment number 17. The figure also includes one minute before and one minute after the sleeping episode. The power values were obtained by the WT analysis and are coded in color (high powers-light tones and low powers-dark tones). **(A)** brain, **(B)** heart.

Complete sleeping episode 3 – crayfish 3

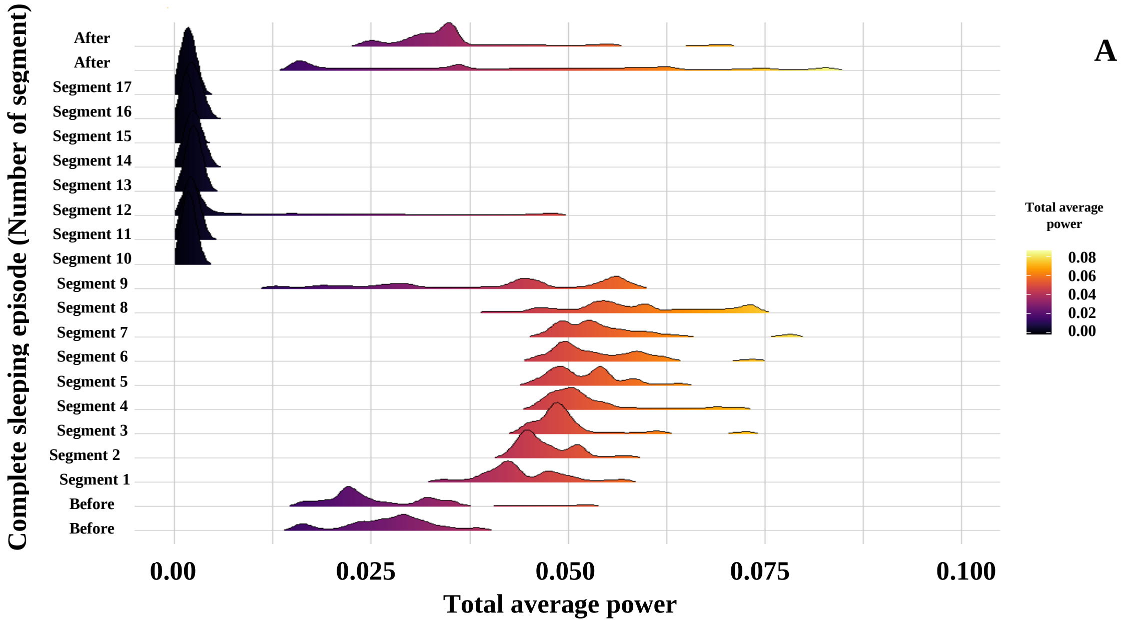

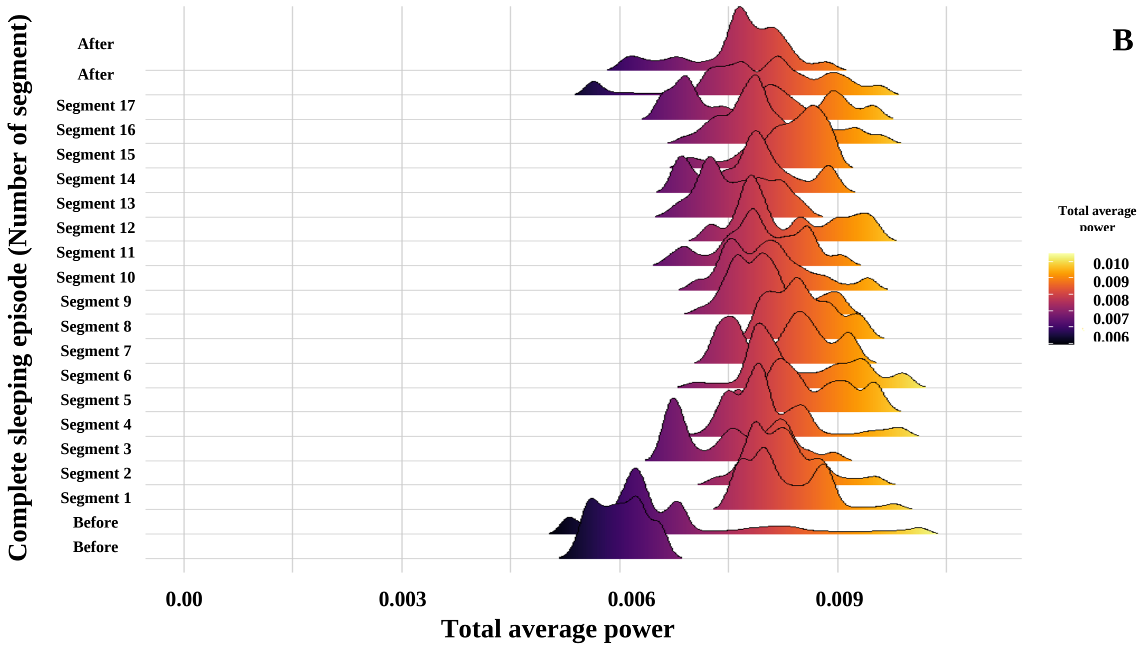

Supplementary. Probability distribution of the total average power of respiratory electrical activity within the frequency band of 0-12 Hz. The graph displays the time evolution of the probability distribution over a complete sleeping episode of about 8.5 minutes from bottom to top. Each segment shows the probability distribution of total average power during 30 seconds windows. The beginning of the sleeping episode corresponds to segment number 1, and it finishes at segment number 17. The figure also includes one minute before and one minute after the sleeping episode. The power values were obtained by the WT analysis and are coded in color (high powers-light tones and low powers-dark tones). **(A)** gill chamber 1, **(B)** gill chamber 2.

Complete sleeping episode 3 – crayfish 3

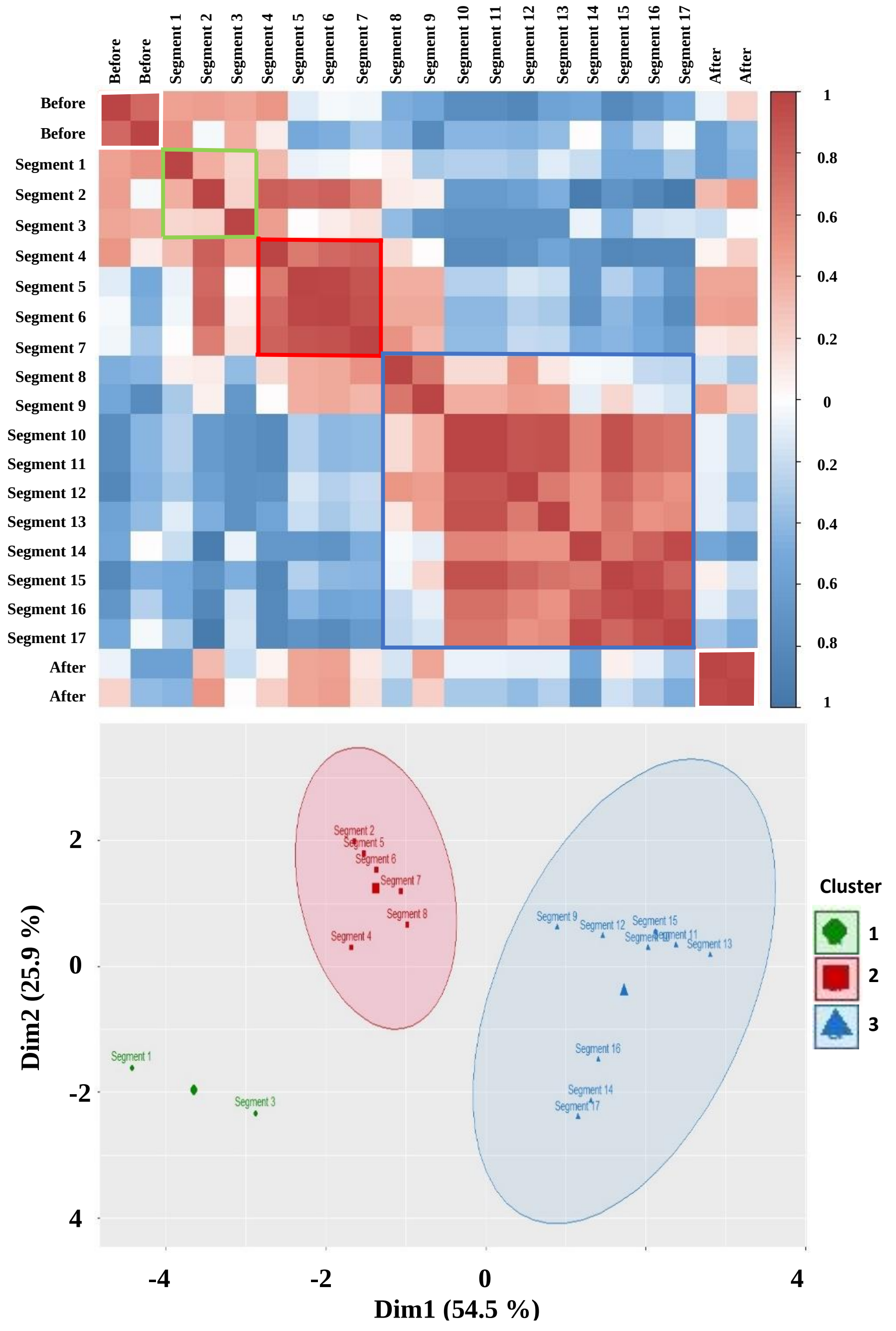

Supplementary. **(A)** Pearson correlation matrix derived from brain and cardiorespiratory electrical activity total average power. This displays the temporal evolution of sleep over a complete sleeping episode of about 8.5 minutes. Each segment shows the coefficients correlation of total average power in~~to~~ 30 seconds windows. The beginning of the sleeping episode is marked with segment number 1, and it finishes at segment number 17. The Figure also includes one minute before and one minute after the sleeping episode (white square). The correlation is coded in color (positive correlations-dark red and negative correlations-dark blue. Each qualitatively identified cluster is framed with a colored square: green square (cluster 1), red square (cluster 2), blue square (cluster 3). **(B)**. *k*-Means clustering corresponding to the same sleeping episode showed in A, each cluster represent a different pattern during sleep. Data are represented by points using principal components, PCA1 explains 54.5 % of total variance and PCA2 explain 25.9 %. A concentration ellipse was drawn around each cluster. Green diamonds (cluster 1), red squares (cluster 2) and blue triangles (cluster3). Results show~~s~~ statistically significant differences between three cluster with medium effect size ($p < 0.05$; $\varepsilon^2 = 0.18$).
